# Machine learning models enhance detection of arrhythmogenic right ventricular cardiomyopathy

**DOI:** 10.1088/3049-477X/ae451a

**Published:** 2026-02-23

**Authors:** Kwaku K Quansah, Sean A Murphy, Esther Kwon, Emma Anderson, Crystal Tichnell, Brittney Murray, Sean Gaines, Alessio Gasperetti, Cynthia A James, Hugh Calkins, Richard T Carrick, Chulan Kwon

**Affiliations:** 1 Department of Cardiology, Johns Hopkins School of Medicine, Baltimore, MD 21202, United States of America; 2 Department of Biomedical Engineering, Johns Hopkins University, Baltimore, MD 21202, United States of America; 3 Cellular and Molecular Medicine Program, Johns Hopkins University, Baltimore, MD 21202, United States of America; 4 Department of Cell Biology, Johns Hopkins University, Baltimore, MD 21202, United States of America; 5 Institute for Cell Engineering, Johns Hopkins University, Baltimore, MD 21202, United States of America; 6 Heart and Vascular Institute, Johns Hopkins Hospital, Baltimore, MD 21202, United States of America

**Keywords:** arrhythmogenic right ventricular cardiomyopathy, machine learning, predictive modeling, gradient boosted trees, matrix clinical data, clinical decision-making, diagnostic support

## Abstract

Arrhythmogenic right ventricular cardiomyopathy (ARVC) is a heritable cardiac disorder associated with sudden cardiac death, yet its diagnosis remains slow, resource-intensive, and dependent on expert interpretation of multimodal tests. Machine learning approaches may enable earlier and more standardized detection. Here, we sought to identify an optimal machine learning strategy for ARVC detection, and to define its role within the diagnostic pathway. A composite dataset of 688 patients from the Johns Hopkins ARVC Registry was used to train and evaluate eight models based on 31 clinical, electrocardiogram (ECG), imaging, and genetic variables. Following an 80/20 train-test split, models were developed on the train set using five-fold stratified cross-validation and evaluated on the hold-out test cohort. Performance was assessed using area under the curve (AUC), accuracy, sensitivity, specificity, and 95% confidence intervals. Model comparison was performed using the Friedman test with Nemenyi post-hoc analysis. Gradient boosted trees (GBT) achieved the highest overall performance (AUC = 0.943, 95% CI: 0.902–0.99; accuracy = 0.847, 95% CI: 0.786–0.907). Nemenyi testing showed GBT significantly outperformed Decision Tree and TabNet (*p* < 0.05), while differences vs the remaining five models were not statistically significant. The next-best model (Random Forest) showed a minimal performance gap (ΔAUC = 0.008), whereas low-ranked models showed larger deficits (ΔAUC = 0.040–0.042). An ECG-only version of the GBT model achieved an AUC of 0.884, exceeding the previously reported ECG-deep learning waveform model (AUC = 0.87). GBT performs best among evaluated algorithms and offers clinically interpretable feature relevance consistent with task force criteria for ARVC diagnosis. An ECG-only deployment supports early triage, while the multimodal model functions as confirmatory decision support after advanced testing. These findings support a tiered ML-assisted diagnostic strategy for ARVC and justify prospective external validation in broader clinical settings.

## 1. Introduction

Arrhythmogenic right ventricular cardiomyopathy (ARVC) is a hereditary structural heart disease characterized by the progressive replacement of right ventricular myocardium with fibro-fatty tissue [1, 2]. This pathological remodeling results in regional wall motion abnormalities and facilitates occurrence of ventricular arrhythmias, thereby elevating the risk of sudden cardiac death (SCD) [3]. Early identification of ARVC is critical to prevent life-threatening ventricular arrhythmias and SCD, which often occur in young individuals and athletes [4, 5]. The arrhythmogenic substrate in ARVC may be present before overt structural changes appear on imaging, and exertion can precipitate malignant arrhythmias in susceptible individuals [6–9]. As a result, timely diagnosis allows for the implementation of lifestyle modification such as activity restriction that may limit disease progression, life-saving interventions such as implantable cardioverter-defibrillator (ICD) placement, and family screening [10–12]. This emphasis on early recognition is reflected in the revised 2010 Task force criteria (2010 TFC), which sought to standardize diagnostic thresholds by incorporating electrocardiographic, imaging, histological, and genetic parameters [13, 14]. Furthermore, the position statement by the Heart Rhythm Society underscores the importance of early detection and risk stratification to guide preventive therapy [15]. Longitudinal data from ARVC registries consistently show that delayed diagnosis is associated with increased arrhythmic burden and worse prognosis, highlighting the need for improved screening tools and early intervention pathways [16–18].

Despite advances in diagnostic criteria, accurately diagnosing ARVC remains a significant clinical challenge [19, 20]. Borderline and gene-elusive presentations often exhibit subtle, variable, or inconclusive findings, leading to high rates of referral for expert re-evaluation [21–23]. Specialized ARVC centers serve as a referral hubs, yet many referred patients ultimately do not meet diagnostic criteria [19, 24]. This places a substantial burden on the limited number of expert centers and also exposes patients to unnecessary interventions, lifestyle restrictions, and psychological distress resulting from misdiagnosis [25]. The high volume of false-positive referrals and the potential for clinical mismanagement highlight the need for more efficient and accurate triage strategies.

Machine learning (ML) and deep learning (DL) approaches can enhance diagnostic precision by identifying complex, non-linear patterns across multimodal clinical data that may be missed by traditional methods [26–28]. Significant effort has been devoted to applying DL models to raw electrocardiogram (ECG) and imaging data to improve diagnostic accuracy [29, 30]. For example, Carrick *et al* recently developed an ECG-based DL (ECG-DL) model that interprets raw ECG waveforms to aid in ARVC diagnosis. While this model achieved a modest but clinically meaningful improvement over expert ECG interpretation (c-statistic 0.87 vs 0.85), diagnostic performance rose substantially (c-statistic 0.94) when ECG-DL outputs were combined with non-ECG clinical features from the 2010 TFC (2010 TFC) [31]. These findings emphasize the added value of multimodal data integration and suggest that future gains in diagnostic accuracy may come more from strategic model design and data fusion than from increasing algorithmic complexity alone.

Building on evidence that diagnostic gains in ARVC arise primarily from effective multimodal integration rather than increasing model complexity alone, our study aims to identify the optimal method for ML based detection of ARVC. Here, we compare alternative ML diagnostic algorithms trained in a large, well characterized cohort of patients referred for ARVC diagnosis at the Johns Hopkins ARVC program.

## 2. Methods

### 2.1. Study population

A retrospective cohort was assembled from the Johns Hopkins ARVC Registry to serve as the basis for algorithm training and internal validation [31]. Patients were included in the cohort if they underwent clinical evaluation for suspected ARVC at Johns Hopkins Hospital (JHH), between January 2011 and September 2019, and had available pre-referral clinical records for review. Inclusion required participation in a standardized second-opinion assessment by a cardiologist with ARVC-specific expertise and a genetic counselor, including review of family history and genetic test results. The Johns Hopkins ARVC Registry is approved by the Johns Hopkins Institutional Review Board, and all data included in this study were collected in accordance with institutional and federal ethical guidelines, with informed consent obtained from all participants.

Due to the sensitive nature of patient-level data, the dataset is not publicly available.

### 2.2. Outcomes

The primary outcome in this study was the fulfillment of the 2010 Modified TFC for the diagnosis of ARVC. The TFC is a standardized, consensus-based diagnostic scoring system composed of major and minor criteria across six categories: (1) global or regional dysfunction and structural alterations, (2) tissue characterization of the ventricular wall, (3) repolarization abnormalities, (4) depolarization/conduction abnormalities, (5) arrhythmias, and (6) family history or genetics.

Each patient was evaluated using clinical, imaging, ECG, and genetic data collected under a uniform institutional protocol. Patients were classified as fulfilling a definite ARVC diagnosis if they met two major criteria, one major plus two minor criteria, or four minor criteria drawn from different categories, in accordance with the 2010 guidelines. All patients were assessed by clinicians following the same standardized diagnostic protocol, and criteria were applied consistently across the dataset. Borderline or indeterminate cases that did not fulfill or clearly fail to meet diagnostic criteria were excluded from model training to avoid label ambiguity. supplemental table 2 summarizes the TFC for diagnosis of ARVC.

### 2.3. Data collection, definition and preprocessing

The initial dataset comprised 688 patient records from the Johns Hopkins ARVC Registry, and feature selection was limited to variables that were readily available in structured tabular form. These features included demographic, genetic, cardiac imaging, and ECG-derived variables relevant to ARVC diagnosis.

Demographic and family history variables contribute contextual and probabilistic information to ARVC diagnosis, supporting risk stratification and interpretation of clinical findings. Demographic and family history variables that were available for this study include Age at first clinical evaluation, Ethnicity, Sex, SCD of first degree relative under the age of 35.

Genetic findings contribute through the identification of pathogenic or likely pathogenic variants in ARVC-associated genes. Genetic variables were encoded as binary features reflecting the presence or absence of a pathogenic variant in ARVC-associated genes, Plakophilin-2 (PKP2), Desmoplakin (DSP), Desmoglein-2 (DSG-2), Desmocollin (DSC), and related genes, to account for the sparse distribution of individual variants within the cohort.

Imaging features derived from echocardiography or cardiac magnetic resonance imaging (MRI) were used to characterize structural and functional cardiac abnormalities. For this study, imaging-derived variables available in tabular form included right ventricular ejection fraction (RVEF) and left ventricular ejection fraction (LVEF) measured by MRI or echocardiography, as well as the presence of late gadolinium enhancement (LGE) on cardiac MRI.

ECG-derived features capture repolarization and depolarization/conduction abnormalities. ECG data acquisition and initial feature derivation for this cohort have been previously described [31]. Briefly, resting 12-lead ECGs were acquired using GE Marquette ECG systems (GE Healthcare, Chicago, IL), with 10 s voltage tracings recorded at a sampling rate of 500 Hz and stored in the GE Muse clinical ECG management system. For the present study, ECGs were not analyzed as raw waveforms. Instead, ECG-derived features were extracted in tabular form based on routine clinical interpretation, including repolarization and depolarization/conduction abnormalities defined according to the 2010 ARVC TFC. These ECG-derived variables were encoded as structured features (e.g. lead-specific T-wave inversion, bundle branch block, terminal activation duration) and incorporated into the multimodal tabular dataset used for model development. Repolarization and conduction features, including *T*-wave inversion, were assessed on resting 12-lead ECGs only; transient ECG changes observed during ambulatory monitoring were not used for feature extraction.

Ambulatory Holter monitoring was performed as part of routine clinical evaluation to assess arrhythmic burden. For the present study, Holter-derived data were used solely to quantify premature ventricular contraction (PVC) burden, expressed as total PVC count per 24 h. Holter recordings were reviewed and summarized in the clinical record by treating cardiologists using standard clinical software. No ECG morphology–based features (e.g., repolarization or conduction abnormalities) were extracted from Holter recordings.

Importantly, ECG features requiring subjective expert interpretation, such as the presence of epsilon waves, were not included in the final feature set. Excluding such features reduced the risk of expert-driven bias and improved the objectivity and reproducibility of the tabular feature set used for ML.

Continuous features (e.g. heart rate, PR interval, LVEF) were standardized to zero mean and unit variance. Binary features (e.g. sex, LGE, bundle branch block, presence of pathogenic variants, presence of *T*-wave inversion in precordial leads) were encoded using 0/1 values. Categorical variables with more than two categories were one-hot encoded.

### 2.4. ML models

Eight ML algorithms were applied to detect ARVC, with hyperparameter tuning performed for most models. These included decision tree, ensemble method-based classifiers (random forests and gradient boosted trees (DBTs)), linear classifiers (Logistic Regression, Lasso Regression, and Ordinary Least Squares (OLSs)) were used for their interpretability and efficiency. A probabilistic classifier, Gaussian Naive Bayes, was included for its capacity to handle high-dimensional data. Additionally, a DL-based model, TabNet, was implemented to explore attention-driven neural network performance on structured, tabular data. Models without hyperparameter tuning (OLSs, standard Logistic Regression, and Naive Bayes) used standard configurations with minor adjustments such as probability clipping or thresholding. Hyperparameter tuning and adjustments for each model are summarized below:

#### 2.4.1. OLSs

OLSs was implemented using a standard linear regression model, adapted for binary classification by thresholding predicted continuous values at 0.5. The model was applied within a 5-fold stratified cross-validation framework. No hyperparameter tuning was performed, as OLS does not have tunable parameters in this context, but predicted values were clipped to the [0,1] range to ensure valid probabilities for ROC and area under the curve (AUC) computation.

#### 2.4.2. Logistic regression

Logistic regression was implemented as a standard classifier for binary outcomes. No hyperparameter tuning was applied to this baseline model, so it was trained using default settings with an intercept term and standard regularization parameters.

#### 2.4.3. Naive bayes

Naive Bayes was implemented without hyperparameter tuning, as the algorithm has very few parameters and is generally robust to default settings. A 0.5 threshold was used to convert predicted probabilities into class labels.

#### 2.4.4. Lasso logistic regression

Lasso Logistic Regression was optimized using GridSearchCV within each fold of 5-fold stratified cross-validation. The hyperparameters explored included the inverse of regularization strength (C), class weighting (class_weight), whether to fit an intercept (fit_intercept), the tolerance for stopping criteria (tol), the solver (solver), the penalty (penalty), and the maximum number of iterations (max_iter). Specifically, the following values were tested:

**Table:** 

Hyperparameter	Description	Values	Best parameters
Regularization strength (C)	Penalty on model complexity	0.001–100	0.1
Class weight	Importance to give to each class to ensure class balance	None, balanced	None
Fit_intercept	Whether to fit an intercept	True, false	True
Tol	Tolerance for the optimization	1 × 10^−4^, 1 × 10^−5^	0.0001
Solver	Numerical optimization method to use to find model coefficients	Liblinear	Liblinear
Penalty	Amount of coefficient shrinkage	L1	L1
Max_iter	Maximum number of iterations	1000, 2000	1000

#### 2.4.5. Decision tree

Decision Tree Classifier was optimized using GridSearchCV. The hyperparameters explored included the maximum depth of the tree (max_depth), the minimum number of samples required to split an internal node (min_samples_split), the minimum number of samples required to form a leaf (min_samples_leaf), and the splitting criterion (criterion). Specifically, the following ranges were evaluated:

**Table:** 

Hyperparameter	Description	Range	Best parameters
Max_depth	Maximum depth of the tree	0–10	3
Min_samples_split	Minimum number of samples required to split an internal node	2–10	2
Min_samples_leaf	Minimum number of samples required to form a leaf	1–4	4
Criterion	Splitting criterion	Gini, entropy	Gini

#### 2.4.6. Gradient boosting trees

Gradient Boosting Classifier was optimized using RandomizedSearchCV for hyperparameter tuning. The hyperparameters explored included the number of boosting stages (n_estimators), the learning rate (learning_rate), the maximum depth of individual regression trees (max_depth), the minimum number of samples required to split a node (min_samples_split) and to form a leaf (min_samples_leaf), the subsample ratio of the training instances (subsample), and the number of features considered for splitting (max_features). The ranges are summarized in the table below:

**Table:** 

Hyperparameter	Description	Range	Best Parameters
n_estimators	Number of boosting stages	50–300	133
Learning_rate	The learning rate	0.01–0.2	0.2
Min_samples_split	Minimum number of samples required to split a node	2–20	3
Min_samples_leaf	Minimum number of samples required to form a leaf	1–10	9
Max_features	Number of features considered for splitting	Sqrt, log2, none	sqrt
Max_depth	Maximum depth of individual regression trees	3–10	8
Subsample	Subsample ratio of the training instances	0.3–0.7	0.77

Continuous and broader ranges were sampled for some hyperparameters, such as n_estimators between 50–300, learning rate between 0.01–0.2, and subsample between 0.7–1.0. and a final model was trained on the full dataset using the most selected hyperparameters across folds.

#### 2.4.7. Random forest

Random Forest Classifier was tuned using GridSearchCV across several hyperparameters to optimize performance. The parameter grid included the number of trees (n_estimators), the maximum depth of each tree (max_depth), the minimum number of samples required to split an internal node (min_samples_split), the minimum number of samples required at a leaf node (min_samples_leaf), and the number of features considered for splitting at each node (max_features). Specifically, the tuning ranges were:

**Table:** 

Hyperparameter	Description	Range	Best Parameters
n_estimators	Number of trees in the forest	50–200	200
Max_depth	Maximum depth of each tree	0–10	10
Min_samples_split	Minimum number of samples to split an internal node	2–10	5
Min_samples_leaf	Minimum number of samples required to be at a leaf node	1–4	2
Max_features	Number of features to consider when looking for the best split	Sqrt, log2, none	Sqrt(no. of features)

Feature importances were extracted and averaged across folds. A final model was trained on the full dataset using the most frequently selected hyperparameters from the cross-validation folds.

#### 2.4.8. TabNet

TabNet is a DL-based architecture designed for tabular data that uses sequential attention to choose which features to reason from at each decision step. For this study, TabNet was implemented using the TabNetClassifier. Hyperparameter tuning was performed using a grid search within each training fold, where a smaller validation set was held out to evaluate candidate configurations. The hyperparameters explored during tuning are summarized in the table below.

**Table:** 

Hyperparameter	Description	Values explored	Best parameters
n_d	Width of the decision prediction layer	8–24	8
n_a	Width of the attention embedding for each step	8–24	16
n_steps	Number of decision steps in the architecture	3–7	5
Gamma	Scaling coefficient for the attention mechanism	1.0–2.0	1.0
Learning_rate	Learning rate for the Adam optimizer	0.005–0.02	0.02
Lambda_sparse	Coefficient for sparsity loss	0–0.01	0
Optimizer_fn	Optimizer function	Adam	Adam
Mask_type	Type of attention mask	Entmax	Entmax
Max_epochs	Maximum number of training epochs	50–100 (tuned per fold)	Varies by fold
Batch_size	Batch size for training	1024	1024
Virtual_batch_size	Virtual batch size for batch normalization	128	128

All model development and analysis were conducted in Python version 3.10, using a suite of open-source libraries tailored for ML applications. We used scikit-learn (v1.2.2) for classical ML algorithms and model evaluation utilities, and PyTorch (v1.13.1) for neural network modeling. The TabNet model was implemented using the pytorch-tabnet library (v3.1.1). Additional libraries used for data processing and visualization included NumPy (v1.24.2) and pandas (v1.5.3). To optimize model performance, hyperparameters for all algorithms except Naive Bayes, Logistic Regression and OLSs, were systematically tuned using GridSearchCV, a grid-based search algorithm from scikit-learn as detailed above.

### 2.5. Model testing and statistics

Model development was performed using an 80/20 train-test split of the dataset, with 80% of the data allocated for training and stratified cross-validation, and the remaining 20% reserved for independent hold-out testing. Categorical variables were summarized as frequencies (%) and compared using Chi-square testing. Continuous variables were presented as mean ± standard deviation or median [interquartile range], and compared using independent sample Students *t*-tests or analysis of variance, respectively. Model discrimination was assessed by calculating the area under the receiver operating characteristic curve using the roc_auc_score function from the scikit-learn library. The classification task was framed as a binary outcome, where the model output represented the predicted probability of a patient meeting diagnostic criteria for ARVC. Continuous probability outputs from each model was dichotomized into ARVC-positive or ARVC-negative classifications using a prediction threshold of 0.5. Probabilities ⩾0.5 were considered indicative of ARVC, while those <0.5 were classified as non-ARVC. This standard cutoff was used to evaluate model performance. A confusion matrix was generated for each model, from which sensitivity, specificity, and overall accuracy were calculated. Sensitivity was defined as the proportion of true positives among individuals with confirmed ARVC, and specificity as the proportion of true negatives among individuals without the disease. Overall diagnostic accuracy was defined as the proportion of all correctly classified cases. Exact binomial confidence intervals (95% CI) were computed for sensitivity and specificity. Performance differences across the eight ML models were assessed using the non-parametric Friedman test, which is appropriate for comparing multiple classifiers evaluated on repeated partitions of the same dataset. The test was applied to the AUC values obtained across the five stratified cross-validation folds, treating each fold as a repeated measure. Post-hoc pairwise comparisons were then conducted using the Nemenyi test to identify which models differed significantly from one another based on their average ranks. Average rank values were computed for all models, and a critical difference (CD) diagram was generated to summarize rank ordering and significance groupings.

## 3. Results

### 3.1. Study cohort characteristics

The study cohort included 688 individuals suspected of having ARVC who were evaluated at the Johns Hopkins ARVC Center between January 2011 and September 2019 [31]. Of these, 358 patients (52%) fulfilled ARVC task-force criteria after comprehensive assessment. Mean age was 39 years, 49% were female and 83% were White. Given the predominance of White patients in the cohort, ethnicity was encoded as a binary variable, with White assigned a value of 1 and all non-White values assigned a value of 0. 525 patients (76%) underwent genetic testing, with pathogenic or likely pathogenic variant identified in 357 patients (68%). On imaging assessment, mean ejection fraction for RV and LV were 41.1% and 55.8% respectively. For the remaining 330 patients who did not fulfill ARVC TFC, alternative diagnoses after comprehensive assessment included vasovagal syncope, idiopathic ventricular tachycardia, idiopathic pvc, athlete’s heart, ventricular fibrillation from ischemia, cardiac sarcoidosis, orthostatic hypotension, myocarditis, tricuspid regurgitation, sinus node dysfunction, atrial fibrillation with aberrancy, left bundle branch block -induced cardiomyopathy, and pectus-induced dyspnea. These patients were assigned the ARVC-negative label for the outcome variable. Demographic, clinical, genetic, and imaging characteristics of the study cohort are summarized in table 1.

**Table 1.** Baseline cohort characteristics for model training and testing. Categorical variables are presented as counts (percentages) and compared using chi-square tests. Continuous variables were assessed for normality using visual inspection and Shapiro–Wilk testing. Normally distributed variables are reported as mean ± standard deviation and compared using independent-sample t-tests, whereas non-normally distributed variables are presented as median [interquartile range] and compared using the Mann–Whitney U test. Measurement units for all continuous variables are provided in table 1.Analyzes were performed using available (non-missing) dta for each variable. TWI = T-wave inversion; PVC = premature ventricular contractions on Holter monitoring; LGE = late gadolinium enhancement; PKP2 = plakophilin-2; DSP = desmoplakin; DSG2 = desmoglein-2; DSC2 = desmocollin-2; CRBBB = complete right bundle branch block; CLBBB = complete left bundle branch block; TAD = terminal activation delay; LVEF = left ventricular ejection fraction; RVEF = right ventricular ejection fraction.

Characteristic (units)	All cases	% Missing	ARVC cases	Non-ARVC cases	*P*-value
Sample size	688		329(47.8%)	359(52.2%)	
Age (years)	40 ± 15	0%	39 ± 15	41 ± 15	0.001
Sex		0%			0.001
Male	394(57.3%)		167(50.8%)	227(63.2%)	
Female	294(42.7%)		162(49.2%)	132(36.8%)	
Ethnicity		0%			0.846
White	576(83.7%)		274(83.3%)	302(84.1%)	
Non-white	112(16.3%)		55(16.7%)	57(15.9%)	
Family screening	138(20.1%)	0%	64(46.4%)	74(53.6%)	0.714
History of SCD in first degree relative <35 years of age	118(17.2%)	0.2%	73(21.6%)	45(12.4%)	0.001
Pathogenic variants in PKP2	152(28.4%)	0%	124(37.7%)	28(7.8%)	<0.001
Pathogenic variants in in DSP	49(7.1%)	0%	33(10%)	16(4.4%)	0.007
Pathogenic variants in DSG2	31(4.5%)	0%	28(8.5%)	3(0.8%)	<0.001
Pathogenic variants in DSC2	13(1.8%)	0%	11(3.3%)	2(0.5%)	0.016
Pathogenic variant in other Gene	35(5%)	0%	22(6.7%)	13(3.6%)	0.098
TWI_V1	391(56.9%)	0.2%	265(80.6%)	126(35.2%)	<0.001
TWI_V2	295(42.9%)	0.2%	238(72.6%)	56(15.6%)	<0.001
TWI_V3	288(41.9%)	0.2%	248(75.4%)	40(11.2%)	<0.001
TWI_V4	205(29.8%)	0.2%	183(55.6%)	22(6.2%)	<0.001
TWI_V5	126(18.3%)	0.2%	115(35%)	11(3.1%)	<0.001
TWI_V6	77(11.2%)	0.2%	72(21.9%)	5(1.4%)	<0.001
TWI_II	16(2.3%)	0.2%	15(4.6%)	1(0.3%)	0.001
TWI_III	35(5.1%)	0.2%	25(7.6%)	10(2.8%)	0.007
TWI_aVF	27(3.9%)	0.29%	20(6.1%)	7(2%)	0.010
CRBBB	49(7.1%)	0.2%	23(7%)	26(7.3%)	1
CLBBB	2(0.29%)	0.3%	0(0%)	2(0.6%)	0.515
LGE	170(34%)	27.3%	133(56.1%)	37(14.1%)	<0.001
TAD	34(5%)	0.2%	24(7.3%)	10(2.8%)	0.011
Heart rate (beats per minute)	65.2 ± 14.4	0%	64.3 ± 15.7	66 ± 13	0.13
PR interval (milliseconds)	159 ± 39	0%	159.4 ± 43.9	158.5 ± 34.2	0.75
QRS duration (milliseconds)	98.9 ± 21.4	0%	97.5 ± 20.8	100.2 ± 21.8	0.1
QT interval (milliseconds)	426 ± 34	0%	431.4 ± 35	421.1 ± 32.2	<0.001
*R*-wave axis (degrees)	39 ± 53	0%	41.8 ± 61.1	35.5 ± 44.9	0.13
LVEF (percentage)	56.7 ± 9.3	12.8%	55.8 ± 10.1	57.48 ± 8.5	0.12
PVC (beats/24 h)	516{0–5288]	15.6%	1963[516–5288]	21[0–1664]	<0.001
RVEF (percentage)	45.3 ± 10.9	31.4%	41.12 ± 10.74	49.10 ± 9.49	<0.001

### 3.2. Feature selection and correlation analysis

The final feature set comprised 31 variables spanning clinical, demographic, genetic, electrocardiographic, and imaging domains relevant to ARVC diagnosis. A comprehensive summary of all features, including definitions, units, value ranges, and the proportion of missing data, is provided in table 2. Missing values were imputed using k-nearest neighbors imputation applied jointly to continuous and binary features following binary encoding. This strategy was chosen to preserve multivariate relationships across clinical, electrocardiographic, genetic, and imaging modalities while minimizing modeling assumptions. Detailed justification, parameter selection, and validation of the imputation approach are described in supplementary methods S1.

**Table 2.** Table of multimodal clinical, imaging, ECG, and genetic features used for model training. Summary of the multimodal feature set used to train machine learning models for arrhythmogenic right ventricular cardiomyopathy (ARVC) detection. Features were derived from diverse clinical modalities, including cardiac MRI, echocardiography, ECG, Holter monitoring, genetics, demographics, and family history. Each variable’s source modality, data type, and encoding scheme are detailed. Inputs spanned binary (e.g., *T*-wave inversion, presence of pathogenic variants), and continuous variables (e.g., heart rate, PR interval, LVEF). The target variable was physician-diagnosed ARVC (1 = ARVC, 0 = non-ARVC). This integrated dataset enabled development of multimodal predictive models leveraging both structural and electrophysiologic cardiac information. *T*-wave inversion was defined as a negative T wave relative to the isoelectric baseline in the specified lead on resting 12-lead ECG, assessed according to standard ECG interpretation and ARVC task force criteria. (supplemental table 2.).

Feature	Feature description	Source modality	Data type	Input	Units
Age at first visit	Age at first clnical evaluation	Demographic	Continuous	Min = 13; Max = 79	Years
Sex	Patient’s biological sex	Demographic	Binary	1 = male; 0 = female	
Ethnicity	Patient’s ethnicity	Demographic	Binary (one-hot encoded)	1 = white; 0 = non-white	
Family screening	Patient presenting with no symptoms and a family history of cardiomyopathy at first assessment	Family history	Binary	1 = Yes; 0 = No	
Istory of SCD in a first degree relative <35 years of age	Sudden cardiac death in first degree relative under the age of 35	Family history	Binary	1 = Yes; 0 = No	
Pathogenic variants in PKP2	Pathogenic variants in PKP2	Genetic	Binary	1 = Yes; 0 = No	
Pathogenic variants in DSP	Pathogenic variants in DSP	Genetic	Binary	1 = Yes; 0 = No	
Pathogenic variants in DSG-2	Pathogenic variants in DSG-2	Genetic	Binary	1 = Yes; 0 = No	
Pathogenic variants in DSC	Pathogenic variants in DSC	Genetic	Binary	1 = Yes; 0 = No	
Pathogenic variants in other genes	Pathogenic variants in other gene	Genetic	Binary	1 = Yes; 0 = No	
Heart rate	Ventricular contractions per minute	ECG	Continuous	Min = 37; Max = 179	Total beats/minute
PR interval	Interval from the *P* wave to the start of the QRS complex	ECG	Continuous	Min = 0; Max = 324	Milliseconds(ms)
QRS duration	Time duration from start of QRS complex to end of *S* wave	ECG	Continuous	Min = 52; Max = 216	Milliseconds(ms)
QT interval	Interval from beginning of Q wave to end of T wave on ECG	ECG	Continuous	Min = 208; Max = 606	Milliseconds(ms)
*R*-wave axis	Angular direction of ventricular depolarization (some deviation is normal)	ECG	Continuous	Range: −87° to +266°	Degrees
TWI_V1	*T*-wave Inversion in precordial lead V1 on resting ECG	ECG	Binary	1 = Yes; 0 = No	
TWI_V2	*T*-wave inversion in precordial lead V2 on resting ECG	ECG	Binary	1 = Yes; 0 = No	
TWI_V3	*T*-wave inversion in precordial lead V3 on resting ECG	ECG	Binary	1 = Yes; 0 = No	
TWI_V4	*T*-wave inversion in precordial lead V4 on resting ECG	ECG	Binary	1 = Yes; 0 = No	
TWI_V5	*T*-wave inversion in precordial lead V5 on resting ECG	ECG	Binary	1 = Yes; 0 = No	
TWI_V6	*T*-wave Inversion in precordial lead V6 on resting ECG	ECG	Binary	1 = Yes; 0 = No	
TWI_II	*T*-wave inversion in II on resting ECG	ECG	Binary	1 = Yes; 0 = No	
TWI_III	*T*-wave inversion in III on resting ECG	ECG	Binary	1 = Yes; 0 = No	
TWI_aVF	*T*-wave inversion in augmented vector foot (aVF)	ECG	Binary	1 = Yes; 0 = No	
CRBBB	Presence of complete right bundle branch block at ECG	ECG	Binary	1 = Yes; 0 = No	
CLBBB	Presence of complete left bundle branch block at ECG	ECG	Binary	1 = Yes; 0 = No	
TAD	Prolonged terminal activation delay/late ventricular activation	ECG	Binary	1 = Yes; 0 = No	Milliseconds(ms)
PVC	Premature ventricular contractions	Holter monitor (ECG)	Continuous	Min = 0; Max = 41 000	Counts/24h
LGE	Late gadolinium enhancement	Cardiac MRI	Binary	1 = Yes; 0 = No	
RVEF	Right ventricular ejection fraction	Cardiac MRI/echo	Continuous	Min = 10; Max = 77.5	Percentage
LVEF	Left ventricular ejection fraction	Cardiac MRI/echo	Continuous	Min = 10; Max = 80	Percentage

**Figure 1.**
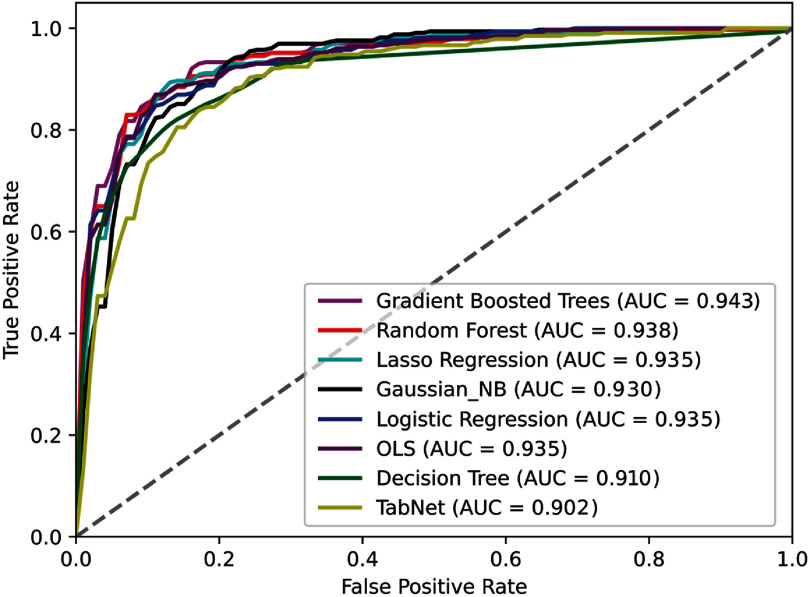
Comparison of ROC of all 8 machine learning models. Comparison of the receiver operating characteristic (ROC) curves of eight machine learning models trained to classify arrhythmogenic right ventricular cardiomyopathy (ARVC) using multimodal clinical, imaging, ECG, and genetic data. The gradient boosted trees model achieved the highest discrimination performance (AUC = 0.943), followed closely by random forest (AUC = 0.938) and Lasso regression (AUC = 0.935). The ROC curves illustrate the trade-off between sensitivity (true positive rate) and 1–specificity (false positive rate) across all models, demonstrating strong overall model performance in distinguishing ARVC-positive from ARVC-negative cases.

**Figure 2.**
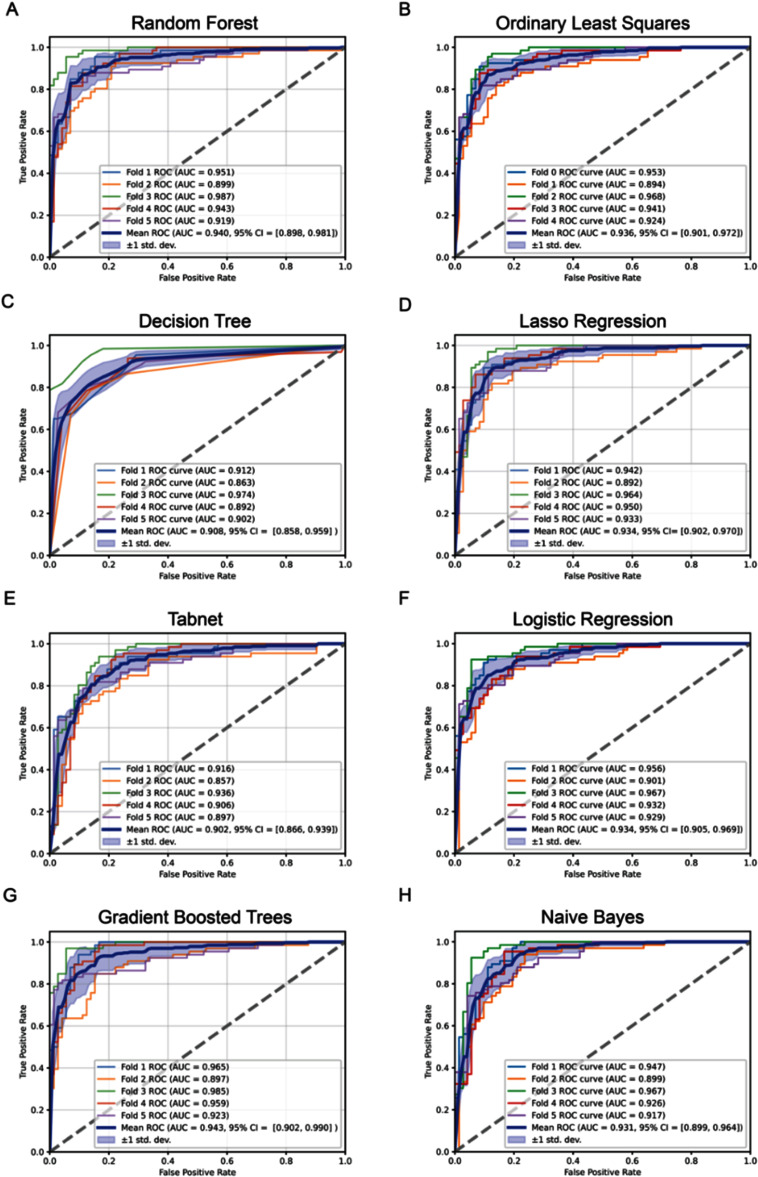
ROC of each trained model A. random forest B. Ordinary least squares C. Decision tree D. Lasso Regression E. Tabnet F. Logistic regression G gradient boosted trees H. Naïve Bayes. This figure shows 5-fold cross-validated ROC curves for all eight classifiers. Each subplot displays fold-specific ROC curves, the mean ROC curve, and its ±1 SD band. Across models, performance is consistently strong (mean AUC ∼0.90–0.96). Gradient boosted trees and random forests perform best, while decision tree and TabNet show slightly lower but still robust AUCs. The figure highlights stable, high discriminative performance across all models. Figure summary of pairwise Pearson correlation coefficients among all input features used for training the ARVC classification models. The matrix highlights inter-feature relationships across multimodal data sources, including ECG, imaging, genetic, demographic, and family history variables. Strong positive and negative correlations indicate features capturing overlapping or inverse physiological information, while low correlations reflect complementary, independent contributions. This analysis was used to assess redundancy, guide feature selection, and ensure model interpretability in downstream training. Feature definition, units, and variable types are described in full detail in table 2.

*Performance of predictive models* GBTs classifier achieved the highest AUC at 0.943, followed closely by Random Forest (0.938) and Lasso Logistic Regression (0.935), suggesting that ensemble-based models and regularized linear methods best captured the underlying feature patterns associated with ARVC diagnosis (figure 1). Both Logistic Regression and OLSs also performed competitively (AUC = 0.935), indicating that linear approaches can achieve performance comparable to more complex architectures. Gaussian Naïve Bayes yielded a slightly lower AUC (0.931) but still demonstrated strong predictive capability. All eight classifiers (random forest, decision tree, TabNet, OLSs, Lasso logistic regression, GBTs, logistic regression, and Gaussian Naïve Bayes) demonstrated strong discriminatory capacity in the hold-out testing cohort, with mean AUC values exceeding 0.90 (figure 2).

Sensitivity and specificity results were similarly strong across models. Lasso logistic regression achieved the highest sensitivity at 0.879 (95% CI: 0.779–0.937), followed by random forest (0.864) and GBTs (0.848). Gaussian Naïve Bayes yielded the highest specificity (0.901; 95% CI: 0.810–0.951), though at the cost of lower sensitivity (0.758). Logistic Regression and OLS produced balanced and moderately high scores, with sensitivities and specificities in the 0.83–0.80 range.

**Figure 3.**
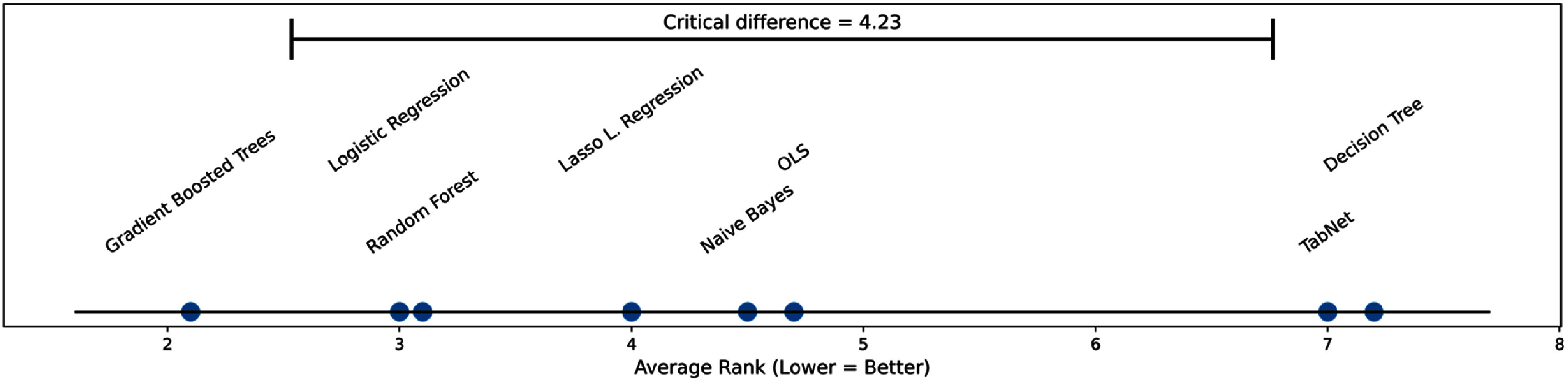
Critical difference (CD) diagram comparing classifier performance based on AUC ranks using the Friedman test with Nemenyi post-hoc analysis. Illustration of the average performance ranks of the eight classification algorithms, where lower ranks indicate better predictive performance. Gradient boosted trees achieved the best average rank (∼2.1), followed by random forest and logistic regression, which cluster near rank 3. Lasso logistic regression, Naive Bayes, and OLS occupy intermediate positions around ranks 4–5. TabNet and decision tree models performed the worst, with average ranks above 7. The horizontal bar at the top represents the critical difference (CD = 4.23); methods whose ranks differ by less than the CD are not statistically significantly different at the selected significance level. Given the width of the CD relative to the spread of ranks, only the very highest-ranked and lowest-ranked methods approach meaningful separation, whereas most models fall within a range where differences are not statistically significant. Overall, the diagram highlights gradient boosted trees as the most consistently high-performing classifier, while TabNet and decision tree show the weakest performance across datasets.

Overall accuracy was consistent across models, ranging from 82.5% to 86.9%. Lasso Regression yielded the highest accuracy (86.9%; 95% CI: 81.2%–92.5%), followed by Random Forest (86.1%) and GBTs (84.7%). All models achieved statistically significant performance with *p* < 0.001 (table 3), underscoring the robustness of the predictive signals learned from the dataset. To evaluate performance beyond raw AUC values, we conducted a non-parametric Friedman test across the five stratified cross-validation folds. The Friedman *χ*^2^ statistic reflects the degree to which the average performance ranks of the eight models deviate from what would be expected if they performed identically, making it an appropriate metric for multi-model comparison [32]. The test confirmed a statistically significant difference in classifier performance (*χ*^2^(7) = 20.30, *p* = 0.004 95), indicating that the models were not statistically equivalent under repeated evaluation. Models were ranked within each fold, and GBTs achieved the best overall average rank (2.10), followed by random forests (3.20) and logistic regression (3.30). In contrast, TabNet (6.80) and Decision Tree (7.20) consistently ranked lowest. (table 4) Direct comparison of mean AUC values relative to GBTs showed small performance differences for competitive models (ΔAUC = 0.008 vs random forest and 0.010 vs Logistic Regression), whereas larger gaps were observed for TabNet (ΔAUC = 0.040) and Decision Tree (ΔAUC = 0.042). Post-hoc Nemenyi testing, summarized in the CD diagram (figure 3), further demonstrated that GBTs significantly outperformed TabNet and Decision Tree, while performance differences between GBTs and the remaining five models were not statistically significant. Thus, although several models achieved high AUC values, GBTs was the only classifier that combined top raw performance with the most favorable and statistically consistent cross-fold ranking.

**Table 3.** Performance comparison of machine learning models for ARVC detection on the test cohort. Table summary of classification performance of eight machine learning models trained to detect arrhythmogenic right ventricular cardiomyopathy (ARVC) using multimodal clinical, imaging, ECG, and genetic data. Model evaluation was performed on an independent test cohort using receiver operating characteristic (ROC) analysis and standard performance metrics. Gradient boosting trees achieved the highest overall performance (AUC = 0.943, 95% CI: 0.902–0.99), followed closely by random forest and Lasso regressor models. Reported metrics include area under the ROC curve (AUC), true negatives (TN), false positives (FP), false negatives (FN), true positives (TP), sensitivity, specificity, accuracy, and associated 95% confidence intervals (CIs). All models demonstrated statistically significant discrimination (*p* < 0.001).

Model	AUC (95% CI)	TN	FP	FN	TP	Sensitivity (95% CI)	Specificity (95% CI)	Accuracy (95% CI)	P-Value
Gradient boosting trees	0.943 (0.902–0.99)	60	11	10	56	0.848 (0.743–0.916)	0.845 (0.743–0.911)	0.847 (0.786–0.907)	<0.00
Random forest	0.938 (0.898–0.981)	61	10	9	57	0.864 (0.761–0.927)	0.859 (0.76–0.922)	0.861 (0.803–0.919)	<0.00
Lasso regressor	0.935 (0.902–0.97)	61	10	8	58	0.879 (0.779–0.937)	0.859 (0.76–0.922)	0.869 (0.812–0.925)	<0.00
Naïve bayes	0.930 (0.899–0.964)	64	7	16	50	0.758 (0.642–0.845)	0.901 (0.810–0.951)	0.832 (0.77–0.895)	<0.00
Logistic regression	0.935 (0.905–0.969)	57	14	10	56	0.848 (0.743–0.916)	0.803 (0.696–0.879)	0.825 (0.761–0.888)	<0.00
OLS	0.935 (0.905–0.969)	60	11	11	55	0.833 (0.726–0.904)	0.845 (0.743–0.911)	0.839 (0.778–0.901)	<0.00
Decision tree	0.910 (0.858–0.959)	62	9	13	53	0.803 (0.692–0.881)	0.873 (0.776–0.932)	0.839 (0.778–0.901)	<0.00
TabNet	0.902 (0.866–0.939)	61	14	10	52	0.788 (0.675–0.869)	0.859 (0.759–0.922)	0.825 (0.761-.888)	<0.00

**Table 4.** Ranking of classifier performance by Friedman test and post-hoc Nemenyi tests. Ranking of eight machine learning classifiers used for ARVC detection based on the Friedman test followed by post-hoc Nemenyi analysis. Lower average rank values indicate superior overall performance across evaluation metrics. The gradient boosted trees model achieved the best mean rank (2.1), followed by random forest (3.2) and logistic regression (3.3), indicating consistent top-tier performance across validation folds.

Models	Average Rank
Gradient boosted trees	2.1
Random forest	3.2
Logistic regression	3.3
Naive bayes	4.3
OLS	4.5
Lasso logistic regression	4.6
TabNet	6.8
Decision Tree	7.2

### 3.3. Feature importance by best performing model (GBTs multimodal model)

An important advantage of GBTs for clinical decision support lies in the algorithm’s interpretability, which can be quantified through feature importance rankings. Two common approaches are: (1) impurity-based importance and (2) permutation feature importance. We elected to use permutation feature importance rather than impurity-based importance because impurity-based measures are biased toward continuous or high-cardinality features and are computed only from training-set splits, which can cause non-informative variables to appear artificially important. In contrast, permutation importance evaluates the decrease in model performance when a feature is randomly shuffled, providing a direct estimate of its contribution to predictive accuracy. To ensure that feature relevance reflected both model learning and true generalizability, permutation importance was calculated on both the training cohort and the independent test cohort. Importance computed on the training set captures the model’s internal dependence on each feature, whereas importance computed on the test set reflects whether that dependence persists on unseen data.

**Figure 4.**
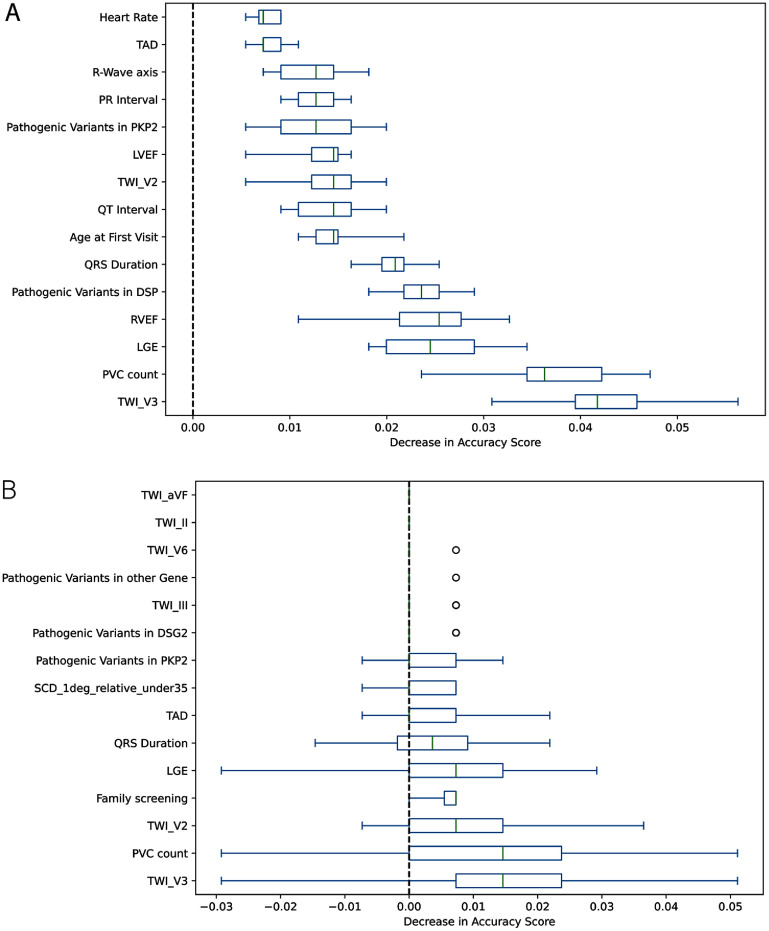
Permutation Importance variability for gradient boosted trees on training and test datasets. Permutation importance variability for gradient boosted trees on (a)*training data:* TWI_V3, PVC count, LGE, RVEF, and pathogenic variants in *DSP* produce the largest decreases in accuracy when permuted, indicating strong contributions. QRS duration, age at first visit, and QT interval show moderate importance, whereas ventricular rate, TAD, and PR interval have minimal effect. (b). *Test data:* feature importances show greater variability but similar patterns. TWI_V3, PVC count, LGE, and TWI_V2 remain the most influential predictors, while genetic variants (*PKP2, DSG2*, and others) retain modest contributions. Several features show near-zero or slightly negative importance, consistent with small-sample variability. Overall, repolarization abnormalities, ventricular ectopy, structural imaging markers, and select pathogenic variants consistently drive model performance across datasets.

**Figure 5.**
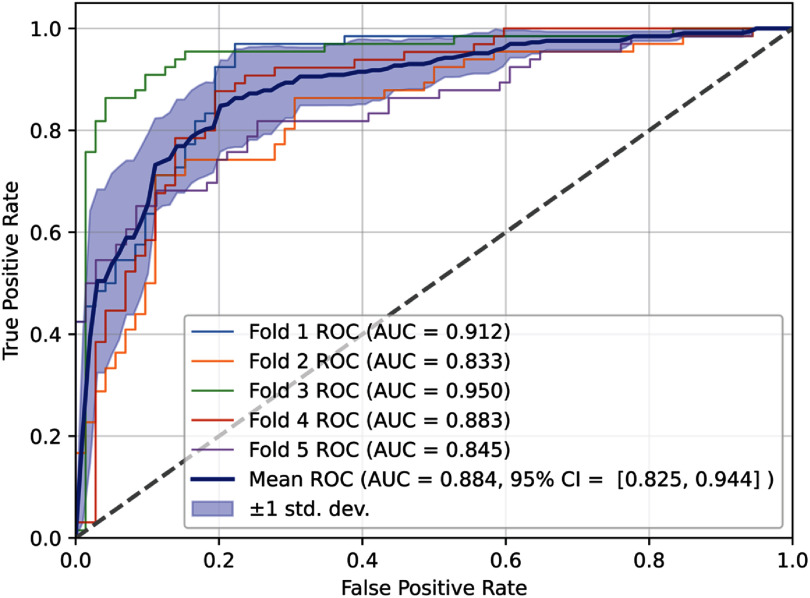
ROC (test data) of gradient boosted trees classifier on ECG-derived features only. Receiver operating characteristic (ROC) curves from five-fold cross-validation of the ECG-only gradient boosted trees model. Individual folds demonstrate consistently strong discrimination (AUC range: 0.833–0.950). The mean ROC curve (blue) yields an overall AUC of 0.884 with a 95% CI of 0.825–0.944, indicating stable performance across folds. The shaded region represents ±1 standard deviation, reflecting modest variability in sensitivity at lower false positive rates and convergence across folds at higher true positive rates.

**Figure 6.**
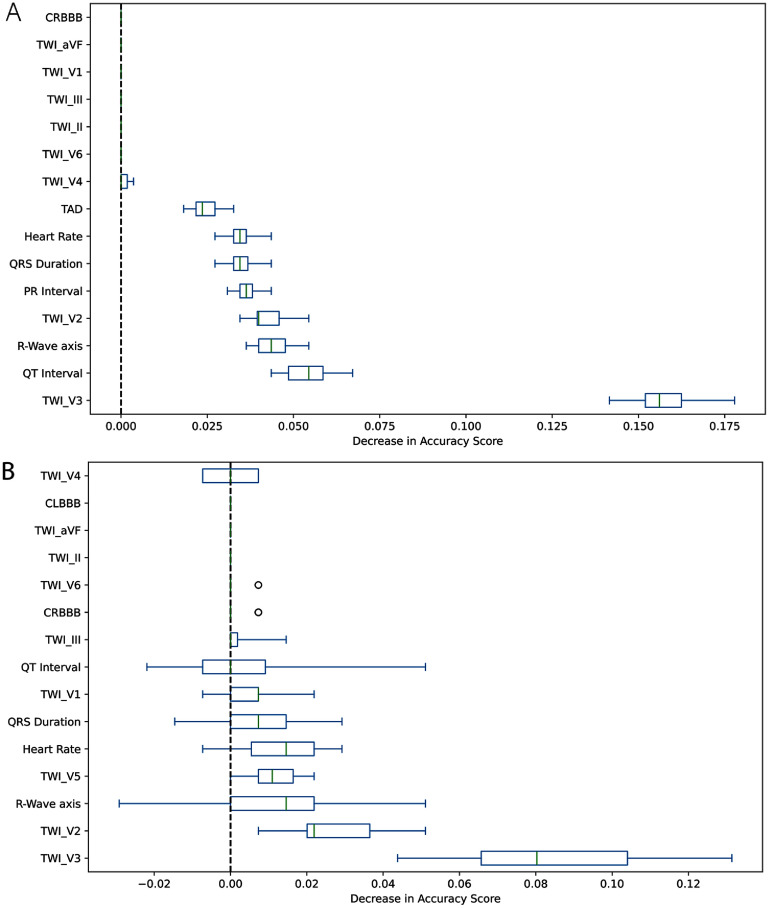
Permutation importance variability for ECG-derived features in the gradient boosted trees model. Permutation importance variability for ECG-derived ARVC diagnostic features in the gradient boosted trees classifier, evaluated on both the training (a) and independent test set (b). Across both datasets, *T*-wave inversion in precordial lead V3 (TWI_V3) is the most influential predictor, producing the largest accuracy drop when permuted. Other consistently important features include TWI_V2, *R*-Wave axis, QT interval, and, to a moderate degree, QRS duration, heart rate, and TWI_V5. Several features, such as CRBBB, CLBBB, and *T*-wave inversion in non-anterior leads, show minimal or near-zero importance. Although test-set variability is higher, the overall ranking of key predictors remains stable, highlighting anterior precordial *T*-wave inversion and axis deviation as the dominant ECG markers driving model performance.

Permutation feature importance analysis of the GBTs model revealed a consistent set of dominant predictors across both the training and independent test cohorts. (figure 4) On the training data, PVC count, *T*-wave inversion in lead V3 (TWI_V3), LGE, and RVEF produced the largest mean decrease in model accuracy when permuted, indicating strong dependence of the model on markers of electrical instability, myocardial fibrosis, and RV dysfunction, three core pathophysiologic domains of ARVC. Additional features with moderate importance included pathogenic variants in PKP2 and DSP, QT and PR interval measurements, and age at first clinical evaluation, reflecting the contribution of both genetic substrate and electrocardiographic remodeling to classification performance. (Figure 5(a)). Evaluation on the held-out test cohort confirmed the stability of these predictors, with PVC count, TWI_V3, LGE, and family history–derived variables (e.g. family screening, SCD in a first-degree relative under age 35) remaining among the highest-impact features. The close correspondence between train and test derived importance rankings supports generalizability and argues against overfitting spurious artifacts in the training set. (Figure 6(b)) Notably, several features exhibited near-zero or negative permutation scores in the test set (e.g., TWI_aVF, TWI_II), suggesting redundancy or limited discriminatory value when evaluated independently of other predictors. Complete summary of full permutation feature importance ranking with mean and standard deviation on both test and train datasets is provided in supplemental table 3. Together, these results demonstrate that the model’s predictive behavior is driven by physiologically plausible and clinically recognized correlates of ARVC, reinforcing the interpretability of the GBTs classifier.

The permutation importance profiles of the GBTs model closely aligned with established ARVC disease mechanisms as defined by the TFC for ARVC diagnosis (supplemental table 2). The highest-impact features on both train and test data were PVC count, *T*-wave inversion in V3 (TWI_V3), LGE, reduced RVEF, and pathogenic variants in desmosomal genes (PKP2, DSP). These features map directly onto three major diagnostic domains of ARVC: arrhythmogenic burden (Category V: >500 PVCs/24 h), ECG repolarization abnormalities (Category III: negative *T* waves in right precordial leads), and structural or fibrofatty myocardial disease (Categories I–II: RV systolic dysfunction and RV/LV LGE). The model’s reliance on pathogenic variants in PKP2 and DSP further reflects Category VI (genetic/family history criteria), which is considered a major diagnostic anchor in contemporary ARVC classification. The concordance between model-derived importance and clinically codified diagnostic determinants supports the face validity of the classifier and indicates that the model is weighting physiologically and diagnostically meaningful signals rather than spurious statistical artifacts. Notably, features outside the core Task Force domains (e.g., non-specific T-wave changes or demographic variables) showed minimal or unstable importance, reinforcing the biological specificity of the model’s predictions.

### 3.4. Comparison with previously published ML models for ARVC detection

To enable a modality-matched comparison with prior studies such as Carrick *et al* [31] and Haq *et al* [33],we trained an ECG-only version of our GBTs model using exclusively ECG-derived features from the same cohort employed in our multimodal models and in Carrick *et al*’s ECG-DL model. ECG-derived features used in training the ECG-only version of the GBTs model is summarized in supplemental table 4. Unlike our approach, which uses interpretable ECG-derived variables (PR interval, QT interval, *T*-wave inversion patterns, bundle branch block status, PVC burden, etc.), Carrick’s ECG-DL model was trained directly on raw 10 s, 12-lead waveform tracings encoded through a variational autoencoder. The ECG-only GBT model achieved a mean AUC of 0.884 (95% CI: 0.825–0.944) (figure 5), similar to the raw waveform ECG-DL model reported by Carrick (AUC = 0.87) and higher than the CNN-based ECG classifier by Haq *et al* (AUC ≈ 0.75) (table 5) However, when extended to a multimodal feature set that included imaging, clinical, and genetic variables, our model achieved a higher AUC of 0.943 (95% CI: 0.902–0.99), comparable to the combined ECG-DL + clinical scoring strategy in Carrick (AUC = 0.940). These results suggest that: (1) ECG alone contains substantial diagnostic signal regardless of whether features are handcrafted or waveform-derived, and (2) multimodal integration yields the strongest discriminative performance when applied to the same patient population.

**Table 5.** Comparison of machine learning models for ARVC detection across published studies and the current work. Comparison between reported AUCs for ARVC detection across prior work and the current study. Earlier ECG-only models ranged from **AUC ∼0.75** Haq *et al* [33] to **0.87** using raw ECG waveforms with a VAE encoder Carrick *et al* [31]. In the current study, an interpretable **ECG-only gradient boosted trees model** achieved **AUC 0.884**. Incorporating non-ECG task force criteria variables improved performance in both studies, with multimodal models reaching **AUC 0.940** Carrick *et al* [31] and **AUC 0.943** in the current gradient boosted trees model.

Study	Input type	Model type	Dataset	Test AUC (95% CI)
Haq *et al* [33]	ECG-derived features	CNN	77 ARVC + matched controls	∼0.75
Carrick *et al* [31]	Raw ECG waveforms (10 s, 500 Hz, 12-lead)	VAE encoder + DL classifier	JHH ARVC registry (same cohort)	0.87 (0.86–0.89)
Current study ECG-only model	ECG-derived features (intervals, TWI, conduction, PVCs)	Gradient Boosted Trees	JHH ARVC registry	0.884 (0.825–0.944)
Carrick *et al* [31])	Raw ECG + non-ECG TFC variables	ECG-DL + clinical scoring	JHH ARVC registry	0.940 (0.933–0.948)
Current study Multimodal GBT	ECG-derived features + non-ECG TFC variables	Gradient Boosted Trees	JHH ARVC registry	0.943 (0.902–0.99)

### 3.5. Comparison of feature importance and clinical applications across multimodal and ECG-only GBTs models

When comparing the feature performance rankings from the multimodal model (figure 4(b)) and the ECG-only model (figure 6(b)), distinct patterns emerge that reflect both the strengths and intended clinical applications of each approach. In the multimodal GBTs model, the highest-ranked features are dominated by structural imaging markers (LGE, RVEF), arrhythmic burden (PVC count), and pathogenic variants (particularly DSP and PKP2), with ECG-derived metrics contributing but ranked lower. These results underscore that ARVC diagnosis in a comprehensive clinical setting is driven primarily by structural remodeling and genetic predisposition, supplemented by electrical abnormalities. Clinically, this model is best suited for full diagnostic evaluations in specialty centers, where imaging data, genetic testing, and detailed clinical assessments are routinely available and can be integrated to maximize diagnostic accuracy.

In contrast, the ECG-only model shows a much narrower distribution of high-impact predictors, relying heavily on anterior *T*-wave inversion (TWI_V3, TWI_V2) and R-Wave axis deviation, with modest contributions from QT interval and QRS duration. Without imaging or genetic inputs, feature importance collapses onto these specific repolarization markers, those most characteristic of ARVC on a surface ECG. Clinically, this model is suited for frontline or resource-limited settings, such as primary care, emergency departments, athletic screening programs, or international contexts where advanced imaging and genetic testing are not immediately available. It provides a lower-cost, ECG-based risk signal that can help triage patients for further evaluation.

Overall, while the multimodal model leverages a broad and clinically diverse set of features for high-precision diagnosis, the ECG-only model provides a parsimonious, accessible tool that identifies key electrical signatures of ARVC when more comprehensive testing is not yet available.

## 4. Discussion

In this study, we evaluated both feature-based and ensemble ML approaches for the detection of ARVC using a multimodal dataset of 688 patients from a high-volume referral registry. Among the eight candidate algorithms, a GBT classifier achieved the highest overall diagnostic performance (AUC = 0.943) and significantly outperformed both traditional linear models and DL approaches when all clinical, ECG, imaging, and genetic variables were available. To clarify the clinical applicability of these models, we additionally developed an ECG-only version of the GBT classifier, which achieved an AUC of 0.884 using routine ECG-derived features alone.

### 4.1. Clinical implications

The present work demonstrates two complementary ML applications within the ARVC diagnostic pathway. First, an ECG-only GBT model provides a low-cost, fully interpretable tool that can be deployed at the earliest stages of evaluation, when no imaging or genomic testing has yet occurred. With an AUC of 0.884, slightly exceeding that of the ECG-DL waveform model reported by Carrick *et al*., this triage-stage model may help identify patients who warrant expedited referral to specialized ARVC centers, reducing diagnostic delay and unnecessary downstream testing.

Second, the multimodal model (ECG + clinical + imaging + genetic features) is intended for use only after standard diagnostic work-up has been completed. Rather than replacing expert interpretation of MRI or genetic testing, the model offers structured, reproducible decision support at the point where clinicians must synthesize heterogeneous data to determine TFC fulfillment. In this setting, the model serves to standardize risk assessment, reduce inter-observer variability, and support diagnostic confidence in borderline or discordant cases.

Future work should prioritize external validation of both model tiers in multi-institutional cohorts with broader demographic and phenotypic diversity. Prospective testing within clinical workflows will be essential to determine whether the ECG-only model improves referral accuracy and whether the multimodal model reduces time to diagnosis or inappropriate deferral. Additional directions include (1) integration of raw waveform and imaging data for hybrid representation learning, (2) longitudinal modeling for prediction of disease conversion or arrhythmic events, and (3) incorporation of emerging genomic features such as polygenic risk scores or transcriptomic signatures, particularly in genotype-negative ARVC.

Together, these staged models, one for early triage and one for definitive diagnostic support, illustrate a scalable and clinically aligned path for ML in inherited arrhythmia syndromes.

### 4.2. Limitations

This study has several limitations that should be acknowledged. First, all data were derived from a single tertiary ARVC referral center, which increases internal consistency but may reduce generalizability to community, screening, or asymptomatic populations. Although the ECG-only model was designed for early triage, both models were trained on a diagnostically enriched cohort, and performance may differ in lower-prevalence settings.

Second, ground-truth labels were based on the 2010 Revised TFC, which do not fully capture newer phenotypic variants, such as left-dominant, gene-elusive, or arrhythmia-first presentations, and future studies should evaluate performance under updated diagnostic frameworks such as the 2020 Padua Criteria or ACM guidelines.

Third, while permutation feature importance supports interpretability, it remains associative and may be affected by collinearity or model-specific interactions. Fourth, although the multimodal model achieved the highest diagnostic performance, it depends on advanced inputs (e.g., LGE, RVEF, genetic testing) that are not universally available, which may limit implementation outside specialty centers unless paired with the ECG-only model in a staged diagnostic workflow.

Finally, genetic features were encoded only as binary indicators of pathogenic variant presence (e.g., PKP2 yes/no) rather than by variant type, location, or pathogenicity class. This simplification does not account for genotype-phenotype variability, variable penetrance, or emerging polygenic effects, and future models incorporating richer genomic information may yield improved performance and mechanistic insight

## 5. Conclusion

This study demonstrates that ML can meaningfully augment the diagnostic workflow for ARVC when aligned with specific points in the clinical pathway. Across eight benchmarked algorithms, GBTs achieved the strongest overall performance on a multimodal dataset integrating ECG, imaging, clinical, and genetic variables (AUC = 0.943), and was statistically superior to several alternative models based on Friedman and Nemenyi tests. A complementary ECG-only version of the model achieved competitive performance (AUC = 0.884) without requiring advanced imaging or genomic data, supporting its potential role as an early triage tool prior to specialist referral.

Permutation-based feature importance confirmed that the model relied on physiologically and diagnostically meaningful predictors, such as PVC burden, *T*-wave inversion, LGE, and desmosomal variants, reinforcing interpretability and consistency with TFC domains. Together, the staged deployment of an ECG-only model for initial risk stratification and a multimodal model for final diagnostic adjudication offers a scalable framework that balances accessibility with diagnostic precision.

Future work should prioritize external validation across diverse populations, incorporation of richer genomic and longitudinal data, and prospective evaluation within real-world clinical settings to determine whether ML-guided support can reduce diagnostic delay, improve standardization of ARVC adjudication, and optimize referral patterns in inherited arrhythmia care.

Overall, these results suggest that ML-assisted ARVC detection can accelerate diagnosis and potentially improve patient outcomes.

## Data Availability

The data cannot be made publicly available upon publication due to legal restrictions preventing unrestricted public distribution. The data that support the findings of this study are available upon reasonable request from the authors. Patient information was not able to be sufficiently deidentified, and is protected. Therefore, the data is not available. Supplementary data 1 available at https://doi.org/10.1088/3049-477X/ae451a/data1.

## Code availability

Code is available on Github at https://github.com/Kwaku-quansah/ml-arvc-detection-pathway.git
